# Crossmodal Modulation of Spatial Localization by Mimetic Words

**DOI:** 10.1177/2041669516684244

**Published:** 2016-12-06

**Authors:** Akihiko Gobara, Yuki Yamada, Kayo Miura

**Affiliations:** Kyushu University, Japan; Japan Society for the Promotion of Science, Japan; Kyushu University, Japan

**Keywords:** spatial localization, representational momentum, mimetic words, mental imagery, multimodal perception

## Abstract

The present study investigated whether aurally presented mimetic words affect the judgment of the final position of a moving object. In Experiment 1, horizontal apparent motion of a visual target was presented, and an auditory mimetic word of “byun” (representing rapid forward motion), “pitari” (representing stop of motion), or “nisahi” (nonsense syllable) was presented via headphones. Observers were asked to judge which of two test stimuli was horizontally aligned with the target. The results showed that forward displacement in the “pitari” condition was significantly smaller than in the “byun” and “nisahi” conditions. However, when non-mimetic but meaningful words were presented (Experiment 2), this effect did not occur. Our findings suggest that the mimetic words, especially that meaning stop of motion, affect spatial localization by means of mental imagery regarding “stop” established by the phonological information of the word.

When observers judge the final position of a moving object, the reported position of the object is displaced forward in the direction of motion. This is called forward displacement (Hubbard, 1996, 2008; Hubberd & Ruppel, 2014; Kerzel, 2002, 2003a, 2003b; Yamada, Kawabe, & Miura, 2010). Previous studies have mainly provided two explanations for this mislocalization phenomenon: a factor of dynamic mental representation based on internalized naive physics and an oculomotor (with attentional) factor.

Numerous previous studies have suggested that the mental representation for a moving object reflects the implied momentum based on the naive knowledge of physics. A moving object with more physical momentum is harder to stop instantly. Analogously, observers forwardly mislocalize the final object position (i.e., representational momentum, RM, Freyd & Finke, 1984). Some RM-related stimulus factors such as velocity (Freyd & Finke, 1985) and acceleration (Finke, Freyd, & Shyi, 1986) of motion have been found to affect forward displacement. Moreover, not only RM but also representational gravity (Hubbard, 1990, 1995, 1997; Hubbard & Bharucha, 1988), friction (Hubbard, 1995; Yamada et al., 2010), and centripetal force (Hubbard, 1996) also affect the displacement of a moving object. These findings suggest that mental representation of a moving object can elicit forward displacement of a moving object.

On the other hand, oculomotor and attentional factors affect forward displacement. For example, when observers fixate on a static point, the forward displacement is dramatically decreased (Kerzel, 2000). This indicates that a combination of pursuit eye movements and visible persistence of a moving object elicits the forward displacement. Moreover, previous studies have reported that observers’ attention to a target affects forward displacement (Hayes & Fryed, 2002; Hubbard, Kumar, & Carp, 2009; Kerzel, 2003a; Müsseler, Stork, & Kerzel, 2002). These studies indicate that both RM and oculomotor or attentional factors are closely related to the forward displacement (see Hubbard, 2005, for a review).

Besides, additional cues also affect forward displacement. For example, when an auditory cue is presented in the display of a moving object, the forward displacement is modulated (Chien, Ono, & Watanabe, 2013; Teramoto, Hidaka, Gyoba, & Suzuki, 2010). Teramoto et al. (2010) showed that a lasting sound from the target’s onset over offset increases forward displacement, and a lasting sound until before the target’s offset decreases it. Chien et al. (2013) demonstrated that a transient sound presented before the disappearance of a moving object weakens forward displacement. These findings indicate that judgments of the final position of visual motion are affected by auditory temporal cues around the target offset, suggesting that visuospatial localization integrates visual motion and auditory temporal signals.

Auditory verbal cues, however, have never been examined as a modulator of forward displacement. Does such a verbal cue affect the forward displacement of a moving object like nonverbal auditory cues (e.g., tone)? The language processing influences visual motion perception such as detection of low-level motion (Meteyard, Bahrami, & Vigliocco, 2007) and motion discrimination (Francken, Kok, Hagoort, & de Lange, 2015; Francken, Meijs, Hagoort, van Gaal, & de Lange, 2015; Pavan, Skujevskis, & Baggio, 2013). In the experiments in these studies, verbs representing directional movement (e.g., sink) were presented before a visual motion stimulus appeared, and the motion verbs congruent or incongruent with visual motion direction affected the error rate and reaction time of motion discrimination (e.g., Meteyard et al., 2007). Moreover, adapting to an aurally presented story including motion verbs also elicits motion aftereffect (Dils & Boroditsky, 2010), indicating that verbal information can cause directional motion adaptation in the visual system. Considering these findings, verbal cues are sufficient to affect visual motion processing, and it is possible that verbal cues bias judgments of the final object position of visual motion by modulating the motion processing.

The present study investigated whether verbal cues affect forward displacement. We used two classes of verbal cues: mimetic words (Experiment 1) and non-mimetic words (Experiment 2). Mimetic words imitate sensory information by their phonological information and contain abundant information based on perceivers’ own sense and experience of motion situations (e.g., “screech”). Indeed, mimetic words contain natural relationships between word sound and meaning (sound symbolism; Hinton, Nichols, & Ohala, 1994). Sound symbolism is empirically demonstrated mainly by the bouba/kiki effect; a pseudoword “bouba” tends to be selected as a name of round figures, and “kiki” tends to be selected as a name of spiky figures (Ramachandran & Hubbard, 2001). This fact indicates that phonological information in sound-symbolic words can elicit the particular mental imagery of shape. Mimetic words may establish mental imagery more vividly than non-mimetic words because non-mimetic words have no particular sound symbolism. Therefore, we predicted that mimetic words would affect the judgment of the final position of a moving object, whereas non-mimetic words would have no or little effect.

## Experiment 1

### Method

Eight Japanese observers (six male and two female, *M* = 23.75) with normal or corrected-to-normal vision participated in this experiment. They received 1,000 Japanese yen for their participation.

All stimuli were presented on a 22-inch CRT monitor (RDF221S; Mitsubishi, Japan). The resolution of the display was 1,024 × 768 pixels, and refresh rate was 100 Hz. Presenting stimuli and collecting data were controlled by personal computer (Mac Pro, Apple, USA). We used MATLAB with Psychotoolbox extension (Brainard, 1997; Pelli, 1997). The viewing distance was 60 cm.

**Figure 1.**
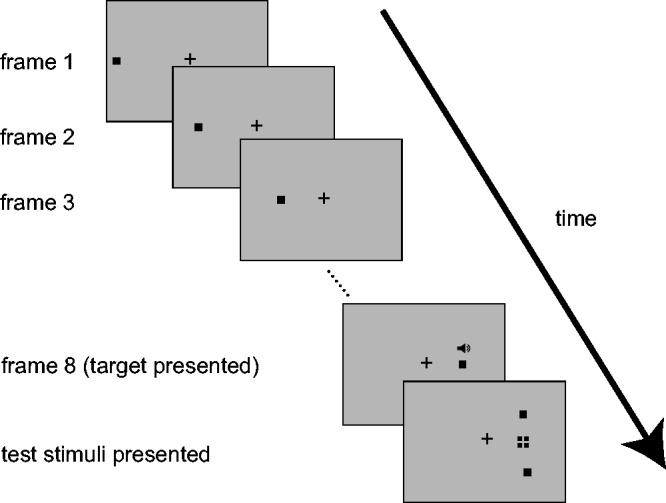
Schematic representation of experiment 1 and 2 (SOA 0 ms condition). The square with a broken line in the frame of test stimuli represents an imaginary vanished position of the target.

Participants began each trial by pressing a spacebar. A fixation point was a black cross presented at the center of the gray background (640 × 480 pixel). Five hundred ms after pressing a spacebar, eight frames of apparent motion of a black square (0.83° × 0.83°) started. The first frame was presented 10.53° left or right of the center, and subsequent frames were presented rightward or leftward (see Figure 1). The horizontal displacement of each square was 1.95°, and the duration and inter-stimulus interval of each square were 30 msec (see Figure 1). The last square was the target to be localized. The horizontal position of the target was randomized within the range of 0.39°. Three hundred ms after the target vanished, two black test stimuli (0.83° × 0.83°) appeared 4° below and above the horizontal median. One was ± 1.4°, ±1°, ±0.6°, ±0.4°, and ±0.2° laterally, and the other was 0° (i.e., the correct test stimulus) relative to the target. (Negative values indicate that the test stimulus was presented backward from the actual vanishing position of the target.) The correct test stimulus was randomized in each trial, and their durations were 100 ms. As a verbal cue, mimetic words or nonsense syllables were presented with three SOAs (−300, 0, and +300 ms; negative values indicate that the verbal cues were presented before the target onset). In each of three word conditions, Japanese mimetic words (“*byun*” mimicking rapid forward motion, “*pitari*” mimicking a stop of motion, or “*nisahi*” as a nonsense syllable) were presented as auditory stimuli via headphones. The durations of these words were 180 ms, 306 ms, and 390 ms, respectively. Participants were instructed to maintain fixation and to judge which of the test stimuli was the same as the last position of the target. After six practice trials, 1,080 experimental trials were conducted in a randomized order.

### Results and Discussion

**Figure 2.**
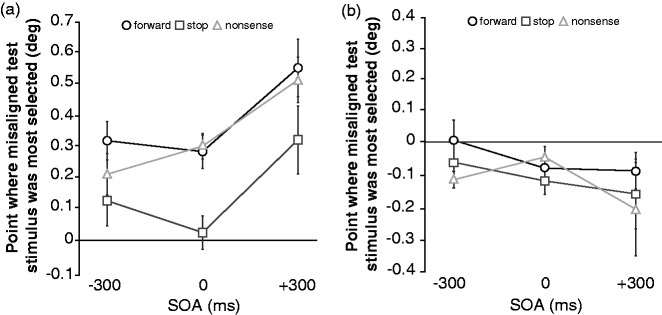
(a) Results of experiment 1. (b) Results of experiment 2. Error bars denote within-participants SEM (Cousineau, 2005).

Fitting a Gaussian function to individual data with the least-square method, the position of the misaligned test stimulus in which error rate was the highest was estimated (Figure 2(a)). An analysis of variance (ANOVA) showed a significant main effect of mimetic words, *F*(2, 14) = 4.42, *p* = .03, η*_p_*^2 ^= 0.39, and no significant main effect of SOA, *F* (2, 14) = 1.14, *p* = .35, η*_p_*^2 ^= 0.14. Multiple comparisons revealed that the estimated position of the misaligned test stimulus in which error rate was the highest in the stop condition was significantly lower than in the forward and nonsense conditions (*ps* < .05). The interaction between mimetic words and SOA was not significant, *F* (4, 28) = 0.34, *p* = .85, η*_p_*^2 ^= 0.05. These results suggest that the mimetic word representing stop of motion significantly affects the forward displacement at least within ± 300 ms of the moving object appearing.

## Experiment 2

### Method

Eight Japanese observers (four male and four female, *M* = 23.38) with normal or corrected-to-normal vision participated in this experiment. They received 1,000 Japanese yen for their participation.

Apparatus, stimuli, and procedure were identical to those in Experiment 1 except that verbal stimuli were Japanese non-mimetic words representing rapid forward motion (“*kousoku*” meaning rapid motion), stop of motion (“*teishi*” meaning a stop of motion), or nonsense syllable (“*nisahi*”). The verbal stimuli were presented aurally, and their durations were 413 ms, 303 ms, and 390 ms, respectively.

### Results and Discussion

The data were analyzed as in Experiment 1. One participant’s data were discarded because the data violated the assumption of normality tested by Shapiro-Wilk test (Shapiro & Wilk, 1965). As in Experiment 1, an ANOVA was conducted (Figure 2(b)), and the result showed that there was no significant main effect of words, *F*(2, 12) = 0.62, *p* = .56, η*_p_*^2 ^= 0.09, SOA, *F*(2, 12) = 0.67, *p* = .53, η*_p_*^2 ^= 0.10, or the interaction between the two, *F*(4, 24) = 0.25, *p* = .90, η*_p_*^2 ^= 0.04. These results suggest that non-mimetic words did not affect the judgments of the final target position.

## General Discussion

In Experiment 1, the mimetic word representing stop of motion significantly decreased the judgments of the final target position, whereas the mimetic word representing forward rapid motion and a nonsense syllable had no effect. However, in Experiment 2, none of the non-mimetic words affected the judgments of the final target position. Moreover, in both experiments, the effect of SOA of the verbal cues was not significant. These results indicate that (a) the phonological information of the mimetic words biases the judgments of the final target position, (b) semantic information cannot solely explain the effect of the mimetic words on the judgments of the final target position, and (c) the effect of mimetic words on the judgment is independent of the cue-target lag at least within the temporal window of ±300 ms.

There are two possible explanations for the effect of mimetic words on the spatial localization of a moving object. First, processing the verbal information of the mimetic words may affect RM. When a mimetic word representing stop of motion (i.e., *pitari*) is presented, the mental representation of stopping is triggered. Thus, this mental representation of stopping may decrease RM. On the other hand, when a mimetic word representing rapid forward motion (i.e., *byun*) is presented, the mental representation elicited by the word does not add any information to the forward stimulus motion, and, hence, the judgment is not affected.

Second, it is possible that capturing more attention by the mimetic word of “stop” decreased forward displacement. When the attention to a moving object is interfered with by a visual distractor, forward displacement decreases (Kerzel, 2003a). A mimetic word representing stop of motion was inconsistent with visual information of the object motion. This incongruence might capture the observer’s attention to the word, thus decreasing forward displacement. On the other hand, the mimetic word of “rapid motion” was congruent with the visual information of the object motion, and hence, observers’ attention might not be captured as much, resulting in no decrement of forward displacement. However, note that the effect of attention on spatial localization varies with previous studies. For example, Hayes and Fryed (2002) argued that dividing attention increases forward displacement while Kerzel (2003a) has claimed that lack of attention to a moving object weakens forward displacement. The attentional explanation for the effect of mimetic words on spatial localization needs to be further investigated in future studies.

Interestingly, non-mimetic words did not affect the judgment of the final target position. This result is inconsistent with previous studies showing that language influences motion perception (Dils & Boroditsky, 2010; Francken, Kok, et al., 2015; Francken, Meijs, et al., 2015; Meteyard et al., 2007; Pavan et al., 2013). Thus, the results of Experiment 2 indicate that mere semantic information of language cannot affect spatial localization even though the word clearly represents stop of motion. Why did only mimetic words bias the judgment? We speculate that the mental imagery formed by phonological information is a key. As mentioned earlier, phonological information of mimetic words is closely associated with mental imagery (Ramachandran & Hubbard, 2001). Considering this, mental imagery based on phonological information of mimetic words may bias the judgment of the final target position. Indeed, imagining an auditory stimulus affected visual stream/bounce judgments (Berger & Ehrsson, 2013). Moreover, phonological information of pseudowords used as an object’s name affects the judgment of the distance to the object (Rabaglia, Maglio, Krehm, Seok, & Trope, 2016). In Experiment 2, only semantic information of non-mimetic words failed to form mental imagery sufficient to bias the judgments of the final target position.

One might argue that the effect of mimetic words derived from a simple response bias like “I heard a stop word, so I am going to respond that the target was at less more forward than with the other words.” The absence of a significant main effect of cue-target SOA might support this claim. However, the results in Experiment 2 excluded this explanation. This is because non-mimetic words should also induce the response bias, but this is not the case. Rather, the non-significance of SOA conditions suggests the relatively large temporal window within which a higher decision level process aggregates information from different sources (i.e., visual motion and auditory verbal stimuli).

There remains an open question of whether visually presented mimetic words also affect visual judgment unimodally. Previous studies have revealed that even visually presented verbal stimuli affect visual motion perception (e.g., Francken, Kok, et al., 2015; Francken, Meijs, et al., 2015). Therefore, mimetic words might bias the judgment of the final target position even unimodally. However, it is difficult to investigate this question because visually presented mimetic words are likely to capture spatial attention. As mentioned earlier, attentional factors affect spatial localization, and thus, disentangling the effect of mimetic words from the effect of attentional capture is very difficult. Moreover, the readability of visually presented verbal stimuli during visual localization tasks should also be involved with this situation. This will be an issue for future research.

In conclusion, in this study, we found that auditory mimetic words did affect the forward displacement of a visually moving target, possibly through the mental imagery based on phonological information of the words, while semantic information itself was not crucial.
